# Responsible and accountable data science

**DOI:** 10.1016/j.patter.2022.100629

**Published:** 2022-11-11

**Authors:** Ben Wagner, Claudia Müller-Birn

**Affiliations:** Department of Technology, Policy and Management, TU Delft; Institute of Computer Science, Freie Universität of Berlin

Over the past few years, there has been an explosion of data science as a profession and an academic field. The increasing impact and societal relevance of data science is accompanied by important questions that reflect this development: how can data science become more responsible and accountable while also responding to key challenges such as bias, fairness, and transparency in a rigorous and systematic manner? This *Patterns* special collection has brought together research and perspective from academia, the public and the private sector, showcasing original research articles and perspectives pertaining to responsible and accountable data science.

The articles in this special collection look closely at both conceptual and practical issues encountered in responsible and accountable data science. From a more conceptual perspective, we have articles looking at how data solidarity could contribute to an understanding of data justice and a review of the ways in which transparency of AI can lead to trust. The conceptual perspective also extends to looking at the data and the algorithms used in data science, with a focus on pointing out data problems around the ways in which data about gender and sex are collected and interpreted and metrics to evaluate the distributional inequality of recommender systems. From a more practice-oriented perspective, the special collection looks at how ethical and legal frameworks influence the work of public sector and police data professionals from the Netherlands, a matrix for auditing automated decision making in hiring, how the regulation of AI is moving toward accountability documentation, and the challenges of AI-based healthcare in the Global South. Finally, there is a valuable contribution on how creating community-based collaborative spaces can contribute to ensuring data ethics become embedded into everyday practices.

Here, we provide a brief overview of the key arguments and perspectives in each of the nine articles in the special collection as well as what we believe is their main contribution to the existing academic debate on responsible and accountable data science.

Braun and Hummel point out that discourses on the notion of justice in the context of data-driven practices tend to focus primarily on conceptions of fairness. In their perspective, they suggest enhancing the notion of data justice with the element of data solidarity. They introduce data solidarity as the commitment to remedy data-facilitated experiences of injustice by integrating shared practices of individuals or groups. Braun and Hummel propose to attend to and act upon the concerns of marginalized groups and to include them in social endeavors mediated by data. Their nuanced conception of data justice and solidarity is a valuable contribution to the field as it emphasizes the responsibility that data scientists and practitioners hold while striving to actively minimize detrimental effects of data science.

In their review, Zerilli, Bhatt, and Weller systematically lay out four categories of transparency that have significance in human-AI (HAI) team coordination: explanations, performance metrics, dynamic allocation strategies, and confidence information. They consider these categories of transparency with regard to their impact on promoting user vigilance. The authors introduce vigilance as an ideal midpoint between algorithm aversion, or distrust, and algorithm appreciation, or overtrust. Importantly, they conclude their review by emphasizing that one of the greatest challenges in the study of HAI teams lies in resisting overgeneralization of experimental results. The authors likewise problematize the effects of “siloed” research, which leads to researchers working on the same subject matter being unaware of discoveries in another field, in part due to unnecessary differences in terminology.

Albert and Delano provide recommendations of how to incorporate sex/gender in medical machine learning variables in such a way that they support nuanced and meaningful findings and avoid common pitfalls. The authors lay out the implications that arise from the issues with sex variables in electronic health data when they are used in data analysis and machine learning in medicine, most notably in terms of the generation of false assumptions and the exclusion of other relevant variables. The recommendations provided by Albert and Delano constitute a valuable contribution to the field of data science and machine learning in the medical context. The authors’ call for deep contextual knowledge that is required to interpret sex/gender variables cannot be emphasized enough.

Looking at the challenges from a more industry-oriented setting from authors working at Twitter, Lazovich et al. attempt to use distributional inequality metrics to measure the degree to which content-recommendation algorithms produce the desired outcomes. The authors are attempting to identify ways in which the production recommendation system at Twitter could be used to ensure that level of engagement with Twitter content is distributed more equitably. Finally, the authors emphasize the need for a more wholistic perspective on metrics, shifting from model-level fairness metrics to system-level fairness metrics.

Looking at public sector and police data professionals in the Netherlands, Fest, Wieringa, and Wagner investigate the influence of ethical and legal frameworks on everyday professional practice. Their study identifies a disconnect between frameworks and practice, partly due to the limits in practicality of the frameworks. The paper contributes to existing debates about accountability and ethics in data science, by arguing for a less principle-based approach. Instead, the paper argues for systematic engagement with key issues across the whole data science project life cycle as well as greater levels of training for both data science professionals and their colleagues without a data science background.

In their perspective on algorithmic decision-making systems (ADSs) used in the hiring domain, Sloane, Moss, and Chowdhury develop a matrix for auditing ADSs that goes beyond technical performance and is aimed at surfacing their underlying assumptions. The sociotechnical matrix that the authors present in their paper can serve as a research tool that helps to identify the concepts that ADSs claim to measure. Most importantly, it also takes into account the underlying assumptions of these concepts that are, as the authors point out, rooted in pseudo-scientific essentialized understandings of human nature and capability.

Okolo contributes to this special collection with a perspective on AI-enabled services in healthcare in the Global South. She problematizes existing practices of AI development that fail to center and prioritize local stakeholders in the process of building technical solutions. Okolo points out the sociotechnical factors that impact successful implementation of AI systems and presents recommendations on how AI and human-computer-interaction practitioners could mitigate potential harms associated with AI solutions for healthcare. Apart from emphasizing the importance of centering the needs of the people operating the systems and encouraging practices of participatory design, Okolo presents a vision for AI in human-centered healthcare. This vision also addresses structural changes, for example with regard to introducing broader impact-statement requirements at premier conference venues.

Oduro, Moss, and Metcalf look in detail at impact assessments as central elements of the governance of automated decision systems in Europe and the United States. They look at commonalities and differences between the Algorithmic Accountability Act of 2022 in the US congress, the New York City’s Int. 1894 from 2021, California’s Assembly Bill 13 (AB 13) from 2021, and European Union AI Act from 2021. The authors conclude that there is a trend toward impact assessments in the regulation of algorithmic systems, which could create “shared ground truths” that could enable other forms of accountability. They urge developers and deployers of automated decision-making systems to prepare for this new reality and ensure they are able to carry out such assessments.

Finally, Di Cara et al. explore how community-based collaborative spaces can contribute to foster quality data science work that is in line with data ethics. They illustrate this effort by example of the Data Ethics Club that they created as a regular and ongoing format for discussing data ethics and how to actively embed this aspect into everyday practice. In their paper, they highlight how data ethics work profits from integrating perspectives from critical work in sociological and philosophical disciplines. Di Cara et al. share their learnings and observations from more than a year of running the Data Ethics Club and provide valuable resources that can be re-used and adapted by others, including an open source reading list. They also provide helpful advice for organizing online group activities that others can build on.

What do we learn from these contributions regarding the advancement of responsible and accountable data science? What becomes clear is that there is no single all-encompassing perspective on what constitutes responsible or accountable data science. Indeed, many of the tools and techniques that would typically be considered central elements of responsibility and accountability, such as ethical and legal frameworks, are struggling to meet the numerous challenges posed by responsible and accountable data science. Instead, there is a great reliance on institutionalizing everyday practices in communities, building auditing and evaluation matrices as well as developing new concepts around solidarity and transparency. There are, however, some new legal frameworks on the horizon in the US and Europe, which may eventually be able to respond to at least some of the existing challenges.

Many of the empirical cases presented in the articles also indicate clear deficiencies in existing responsible and accountable data science practices, with harm being caused to healthcare patients in the Global South (Okolo). These harms can often be traced back to conceptual failures related to the way in which data are interpreted or algorithmic systems are built and interpreted. Rarely is the harm caused by errors in highly advanced machine learning systems. More typically, the broader conceptual errors take place in simpler algorithmic systems.

Another common thread is the lack of sufficient training of staff using algorithmic systems, which would be a prerequisite to ensuring meaningful interpretation of system output. This lack of training is particularly challenging in the case of public sector professionals who often deal with vulnerable populations. Consistent with this, the papers in this special collection point to the urgent need for more training for public sector professionals working with data and algorithms and even more so for those dealing with vulnerable populations.

More broadly, the papers demonstrate a community of data scientists across a wide variety of different professional contexts, striving toward finding better solutions to the challenge of responsible and accountable data science. Their responses, ranging from conceptual to practical, all tackle a different angle of the problem, yet not a single paper suggests that any of their ideas would even come close to solving the myriad challenges they have encountered.

However, there is still much crucial but incremental work to be done to ensure more responsible and accountable data science. In consequence, in consultation with the *Patterns* editors, we have decided to turn this special collection into a live special collection. There are far too many open questions to simply finish this editorial and walk away. Therefore, we continue to welcome submissions for this special collection, which is now a live special collection. Once sufficient additional articles have been received, we will provide a novel editorial perspective to provide an updated overview of this collection.

Topics of the special collection include but are not limited to:

• Research and perspectives on increasing the responsibility and accountability of data science in high-risk environments (healthcare, national security and intelligence, public services, elections, humanitarian aid and development)
• Regulatory and ethical frameworks that can contribute to the development of more responsible data science practices that promote accountability
• Approaches toward developing responsibility-by-design and accountability-by-design systems and mechanisms for data science
• Research and perspectives on what human-centered data science could look like in practice
• Research and perspectives on systematic ways of presenting the data scientists’ results that emphasizes their situatedness, limitations, uncertainty, and bias

